# Identity Determinants of the Translocation Signal for a Type 1 Secretion System

**DOI:** 10.3389/fphys.2021.804646

**Published:** 2022-02-10

**Authors:** Olivia Spitz, Isabelle N. Erenburg, Kerstin Kanonenberg, Sandra Peherstorfer, Michael H. H. Lenders, Jens Reiners, Miao Ma, Ben F. Luisi, Sander H. J. Smits, Lutz Schmitt

**Affiliations:** ^1^Institute of Biochemistry, Heinrich Heine University Düsseldorf, Düsseldorf, Germany; ^2^Center for Structural Studies, Heinrich Heine University Düsseldorf, Düsseldorf, Germany; ^3^Department of Biochemistry, University of Cambridge, Cambridge, United Kingdom

**Keywords:** bacterial secretion systems, secretion signal, ABC transporter, amphipathic helix, ATPase activity, protein secretion

## Abstract

The toxin hemolysin A was first identified in uropathogenic *E. coli* strains and shown to be secreted in a one-step mechanism by a dedicated secretion machinery. This machinery, which belongs to the Type I secretion system family of the Gram-negative bacteria, is composed of the outer membrane protein TolC, the membrane fusion protein HlyD and the ABC transporter HlyB. The N-terminal domain of HlyA represents the toxin which is followed by a RTX (Repeats in Toxins) domain harboring nonapeptide repeat sequences and the secretion signal at the extreme C-terminus. This secretion signal, which is necessary and sufficient for secretion, does not appear to require a defined sequence, and the nature of the encoded signal remains unknown. Here, we have combined structure prediction based on the AlphaFold algorithm together with functional and *in silico* data to examine the role of secondary structure in secretion. Based on the presented data, a C-terminal, amphipathic helix is proposed between residues 975 and 987 that plays an essential role in the early steps of the secretion process.

## Introduction

Type 1 secretion systems (T1SS) are widespread in Gram-negative bacteria and translocate a large variety of mainly proteinaceous substrates (Holland et al., 2016). The general blueprint of such a nanomachinery consists of an ABC transporter, a membrane fusion protein (MFP) and an outer membrane protein (OMP). In the presence of a substrate, the three components form a continuous channel across the inner and outer membrane, which allows the translocation of the substrate from the cytosol into the extracellular space in one step.

A well-known member of sub-family 2 of T1SS is the hemolysin A (HlyA) T1SS, which is composed of the ABC transporter HlyB, the membrane fusion protein HlyD and the outer membrane protein TolC [for recent reviews see Kanonenberg et al. (2013)], which was first identified in uropathogenic *E. coli* strains (Felmlee et al., 1985). The secretion signal of the substrate is located at the extreme C-terminus and is not cleaved prior, during or after transport (Gray et al., 1986). Additionally, these substrates are characterized by Gly- and Asp-rich nonapeptide repeats, the so-called GG-repeats (Welch, 2001). These GG-repeats with the consensus sequence GGxGxDxUx (x: any amino acid, U: large, hydrophobic amino acid) bind Ca^2+^ ions with an affinity of approximately 150 μM (Sanchez-Magraner et al., 2007). As the concentration of free Ca^2+^ ions in the cytosol is around 300 nM (Jones et al., 1999), orders of magnitude below the K_D_, substrates of sub-family 2 remain unfolded in the cytosol, as demonstrated for HlyA (Bakkes et al., 2010). In contrast, Ca^2+^ concentration in the extracellular space is around 2 mM. This results in binding of Ca^2+^ ions to the GG-repeats, which induces folding of the entire protein and formation of a β-roll structure similar to that first identified in *Pseudomonas aeruginosa* alkaline protease (Baumann et al., 1993). The GG repeats in the β-roll defines the Repeat in ToXins (RTX) domain that is found in a large family of T1SS-secreted proteins, and these are accordingly referred to as RTX proteins.

With the exception of sub-family 1 (Kanonenberg et al., 2013), all other substrates of T1SS contain a C-terminal secretion signal at the extreme C-terminus that is necessary and sufficient for secretion (Mackman et al., 1987). Mutational studies of HlyA revealed that the secretion “information” is located in the last 50 to 60 residues (Nicaud et al., 1986; Mackman et al., 1987; Koronakis et al., 1989; Jarchau et al., 1994). However, despite extensive research, the exact nature of the information or code remains enigmatic. Based on sequence comparisons, no real conservation on the level of primary structure was evident within all sub-families (Holland et al., 2016). This was confirmed by random mutagenesis of the secretion signal of HlyA, indicating a high level of redundancy with only eight positions showing drastic influences on secretion efficiencies (Kenny et al., 1994). This redundancy led to the proposal that secondary structures might be encoded in the secretion signal. A putative amphipathic α-helix located between residues 973 and 987 of HlyA was first proposed by *in silico* approaches and subsequently supported by mutagenesis studies (Koronakis et al., 1989; Stanley et al., 1991) that indicated a larger α-helix between residues 976 and 1001. However, the presence of such a helix remained under debate, and a series of studies either supported or contradicted the theory (Stanley et al., 1991; Kenny et al., 1992, 1994; Chervaux and Holland, 1996). A combinatorial approach combined with structural studies provided further support for the importance of an amphipathic helix, now situated between residues 975 and 988 (Yin et al., 1995; Hui et al., 2000; Hui and Ling, 2002). Unfortunately, the crystal structure of the C-terminal part of the RTX domain of CyaA did not provide further information, as the last 33 C-terminal amino acids covering the corresponding region in CyaA were disordered in the structure (Bumba et al., 2016). Thus, the nature of the code of the secretion signal is still unclear, and it is also an open question whether all RTX proteins use the same code to initiate secretion: secondary structure predictions as well as the few crystal structures of proteases and lipases of the RTX family indicate rather the presence of β-strand structures, but not an α-helical content of the C-terminus (Baumann et al., 1993; Meier et al., 2007).

In this study, we re-examined the role of C-terminal secretion signal of HlyA based on a set of mutants (Chervaux and Holland, 1996) within the proposed amphipathic α-helix, but extended the number of mutants by including proline residues. Since the hemolytic activities of all mutants were not affected, we focused on the initial steps of secretion and determined the rate of secretion per transporter. Here, important differences became apparent pointing toward an essential role of a putative amphipathic α-helix in the secretion of HlyA. Additionally, we further supported the hypothesis by an *in silico* analyses of the primary sequence and by modeling the structure of HlyA using AlphaFold (Jumper et al., 2021). Overall, our results strongly support the essential role of this amphipathic α-helix in the initiation step of the secretion process of HlyA.

## Materials and Methods

### AlphaFold Prediction of the HlyA Structure

AlphaFold (Jumper et al., 2021) was used to predict the structure of HlyA (Uniprot entry P08715) employing the ColabFold web interface^1^ using standard settings (five models and no templates).

### Cloning of Pro-HlyA Mutants

Mutations were introduced in the pro-HlyA plasmid pSU-HlyA (Thomas et al., 2014a) by applying the quick-change PCR method using primers listed in Table 1 and following the protocol of the manufacturer (New England Biolabs).

**TABLE 1:** Primers used for quick-change polymerase chain reaction.

Mutant	Forward primer	Reverse primer
P975G	CAGGGTGATCTTAATGGAT TAATTAATGAAATCAGC	GCTGATTTCATTAATTAA TCCATTAAGATCACCCTG
N978G	GATCTTAATCCATTAATTGG TGAAATCAGCAAAATC	GATTTTGCTGATTTCACC AATTAATGGATTAAGATC
E979G	CCATTAATTAATGGAATCA GCAAAATCATTTCAGCTGC	GCAGCTGAAATGATTTTGC TGATTCCATTAATTAATGG
E979P	CCATTAATTAATCCAATCA GCAAAATCATTTCAGCTGC	GCAGCTGAAATGATTTTGC TGATTGGATTAATTAATGG
I980S	CCATTAATTAATGAATCCA GCAAAATCATTTCAGCTGC	GCAGCTGAAATGATTTTGC TGGATTCATTAATTAATGG
I980P	CCATTAATTAATGAACCCA GCAAAATCATTTCAGCTGC	GCAGCTGAAATGATTTTGC TGGGTTCATTAATTAATGG
S981I	CATTAATTAATGAAATC ATCAAAATCATTTCAGC	GCTGAAATGATTTTGATG ATTTCATTAATTAATG
S981P	CATTAATTAATGAAATCC CCAAAATCATTTCAGCTG	CAGCTGAAATGATTTTGG GGATTTCATTAATTAATG
K982T	CCATTAATTAATGAAATCA GCACAATCATTTCAGCTGC	GCAGCTGAAATGATTGTG CTGATTTCATTAATTAATGG
K982P	CCATTAATTAATGAAATCAG CCCAATCATTTCAGCTGC	GCAGCTGAAATGATTGGGC TGATTTCATTAATTAATGG
I983S	GAAATCAGCAAAAGC ATTTCAGCTGCAG	CTGCAGCTGAAATG CTTTTGCTGATTTC
I984S	GAAATCAGCAAAATC AGCTCAGCTGCAGG	CCTGCAGCTGAGCTG ATTTTGCTGATTTC
I984P	GAAATCAGCAAAATC CCTTCAGCTGCAG	CTGCAGCTGAAGGGAT TTTGCTGATTTC
S985A	CAGCAAAATCATTG CAGCTGCAGG	CCTGCAGCTGCAA TGATTTTGCTG
S985P	CAGCAAAATCATTC CAGCTGCAGG	CCTGCAGCTGGAA TGATTTTGCTG
F990P	CATTTCAGCTGCAGGTAGCC CCGATGTTAAAGAGGAAAG	CTTTCCTCTTTAACATCGGGG CTACCTGCAGCTGAAATG
I983P	GAAATCAGCAAACC CATTTCAGCTGCAG	CTGCAGCTGAAATG GGTTTGCTGATTTC
A986P	GCAAAATCATTTCA CCTGCAGGTAGC	GCTACCTGCAGGT GAAATGATTTTGC
E979G-I980S -K982T	CCATTAATTAATGGATCCAG CACAATCATTTCAGCTGC	GCAGCTGAAATGATTGTGC TGGATCCATTAATTAATGG
E979G-K982T	CCATTAATTAATGGAATCA GCACAATCATTTCAGCTGC	GCAGCTGAAATGATTGTGC TGATTCCATTAATTAATGG
E979P-I980P -K982P	CCATTAATTAATCCACCCA GCCCAATCATTTCAGCTGC	GCAGCTGAAATGATTGGG CTGGGTGGATTAATTAATGG
E979G-I980S	CCATTAATTAATGGAAGCA GCAAAATCATTTCAGCTG	CAGCTGAAATGATTTTGC TGCTTCCATTAATTAATGG
I980S-K982T	CCATTAATTAATGAAAGCA GCACAATCATTTCAGCTGC	GCAGCTGAAATGATTGTG CTGCTTTCATTAATTAATGG

### Overexpression and Purification of Pro-HlyA and Mutants From Inclusion Bodies

Overexpression and purification was performed as described in Thomas et al. (2014a). In brief, the expression of pro-HlyA was induced by adding 1 mM IPTG to cultures. Incubation was continued for 4 h and cells were harvested by centrifugation (8000 g, 10 min, 4°C). For the purification of pro-HlyA, cells were resuspended in 50 mM HEPES pH 7.4, 150 mM NaCl, 10% (w/v) glycerol, 0.05% (w/v) NaN_3_ and lysed by passing three times through a cell disruptor at 1.5 kbar (M-110P, Microfluidics). Inclusion bodies were collected by centrifugation at 18,000 g for 30 min. Pellets were washed and centrifuged successively in (1) 50 mM HEPES, pH 7.4, 50 mM EDTA, 1% (w/v) Triton X-100, 0.05% (w/v) NaN_3_ and (2) 50 mM HEPES, pH 7.4, 1 mM EDTA, 1 M NaCl, 0.05% (w/v) NaN_3_. The pellet was solubilized overnight in 20 mM HEPES pH 7.4, 20 mM NaCl, 6 M urea) at room temperature. Insoluble material was removed by ultra-centrifugation (150,000 g, 30 min, 4°C) and the urea-solubilized inclusion bodies were stored at −80°C.

### Small Angle X-ray Scattering Measurements

Size exclusion chromatography coupled small angle x-ray scattering (SEC-SAXS) data of refolded proHlyA were collected on beamline BM29 at the ESRF Grenoble (Pernot et al., 2010, 2013). The BM29 beamline was equipped with a PILATUS 1M detector (Dectris) at a fixed distance of 2.869 m. The measurement of refolded pro-HlyA (8.0 mg/ml, 110 μL inject) were performed at 10°C on a Superose 6 increase 10/300 column, preequilibrated with 100 mM HEPES pH 8.0, 250 mM NaCl, 10 mM CaCl_2_. with a flowrate of 0.5 ml/min, collecting one frame every 2 s. Data were scaled to absolute intensity against water.

All programs used for data processing were part of the ATSAS Software package (Version 3.0.4) (Manalastas-Cantos et al., 2021). Primary data analysis was performed with the program CHROMIXS (Panjkovich and Svergun, 2017) and PRIMUS (Konarev et al., 2003). With the Guinier approximation (Guinier, 1939), the forward scattering *I(0)* and the radius of gyration (*R*_*g*_) were determined. The program GNOM (Svergun, 1992) was used to estimate the maximum particle dimension (*D*_*max*_) with the pair-distribution function *p(r)*. Low resolution *ab initio* models were calculated with GASBORMX (Svergun et al., 2001; Petoukhov et al., 2012) (P2 Symmetry). Dimer docking of the calculated AlphaFold (Jumper et al., 2021) monomer model was done with SASREFMX (Petoukhov and Svergun, 2005; Petoukhov et al., 2012). Superimposing of the calculated dimer model was done with the program SUPCOMB (Kozin and Svergun, 2001). The monomer/dimer content of the scattering data was determined with OLIGOMER (Konarev et al., 2003) using the AlphaFold monomer and the SASREFMX dimer as input.

### *In vitro* Acylation Assay and Hemolytic Activity of HlyA

An *in vitro* acylation protocol was applied as described in Thomas et al. (2014b). Briefly, the pro-HlyA mutants were unfolded in 6 M urea and any divalent cations were removed by adding 10 mM EDTA. Pro-HlyA was mixed with HlyC and acyl-carrier protein (ACP) and the hemolysis-efficiency on erythrocytes was quantified by measuring the hemoglobin release at 544 nm (Thomas et al., 2014b) at a final concentration of HlyA of 18 μg/ml (160 nM). 1 μl of 16% SDS solution in 74 μl assay was used as positive control to determine the value of 100% cell lysis. The concentration of wildtype HlyA and the mutants was chosen as it represents the lowest concentration of wildtype HlyA with the highest lytic activity (Thomas et al., 2014b).

### Secretion Assay of Pro-HlyA-Mutants

The secretion rate of the pro-HlyA mutants was determined as described before (Lenders et al., 2016). Briefly, cells were grown for a total of 4 h. Every hour, samples were taken and the supernatants were analyzed by SDS-PAGE. Pro-HlyA as well as the secretion apparatus was subsequently quantified and the secretion rates were determined as amino acids per second and transporter as described in detail in (Lenders et al., 2016).

### Secondary Structure Prediction

Quick2D (Zimmermann et al., 2018) and AmphipaSeeK (Combet et al., 2000; Sapay et al., 2006) were used to predict the secondary structures. Quick2D is able to predict α-, π- and TM-helices, β-strands, coiled coils, as well as disordered regions (Zimmermann et al., 2018). AmphipaSeeK, on the other hand, is specifically designed to identify amphipathic helices (Sapay et al., 2006). The output includes a secondary structure prediction, a predicted membrane topology (in-plane or not in-plane), a prediction score for the proposed membrane topology and an amphipathy score for each residue in dependence to the neighboring residues.

### Structure Prediction of HlyA Derived Peptides

PEP-FOLD3 was used to model peptides of HlyA (Thevenet et al., 2012; Shen et al., 2014; Lamiable et al., 2016).

### Illustration and Visualization

The amphipathic nature of a helix was visualized by a helical wheel projection using NetWheel (Mol et al., 2018). Protein and peptide structures were visualized using PyMOL.^2^ In order to illustrate and identify hydrophobic surfaces the yrb-script was applied in PyMOL, which highlights carbon atoms that are not bound to oxygen or nitrogen in yellow, the charged oxygens of Glu and Asp residues in red, the charged nitrogens of Lys and Arg residues in blue, while all other atoms are white (Hagemans et al., 2015).

## Results

**FIGURE 1:**
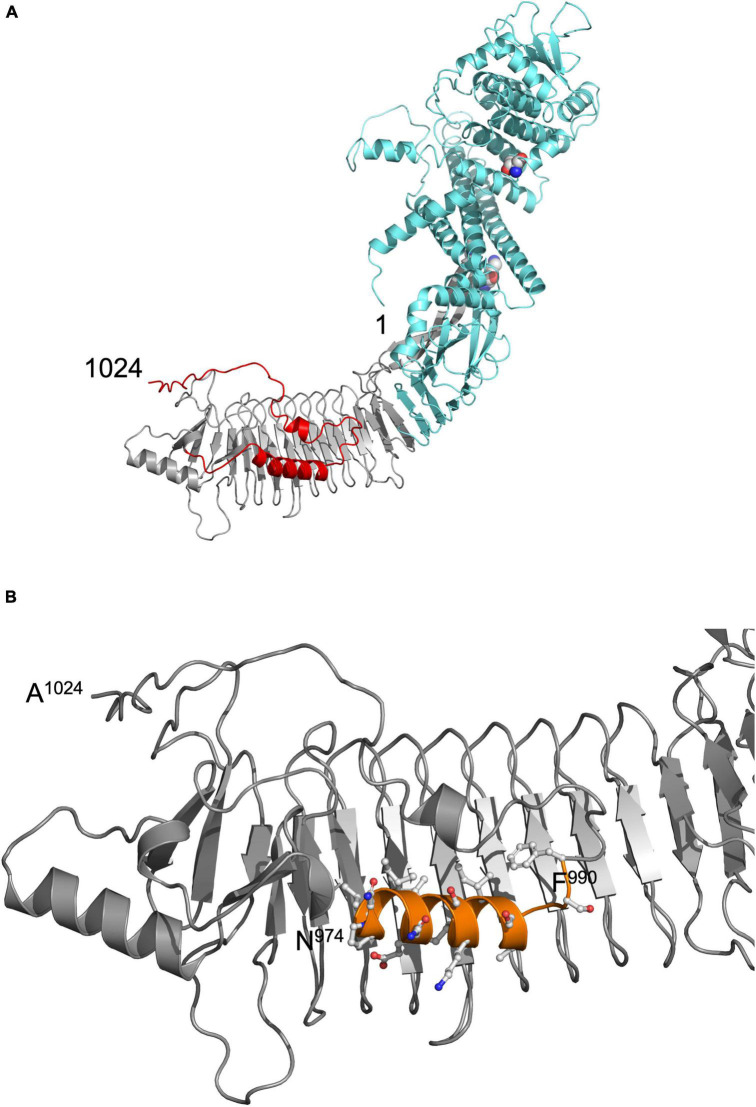
**(A)** Model of pro-HlyA predicted by AlphaFold (Jumper et al., 2021). The N- and C-termini are indicated by numbers (1 and 1024, respectively). The secretion signal is highlighted in red, the RTX in gray and the N-terminal pore-forming domain in cyan. The two Lys residues (K564 and K690) that are acylated by HlyC prior to secretion are highlighted as spheres. **(B)** Zoom in into the C-terminal region of the structural model of folded pro-HlyA. The C-terminus (A^1024^) as well as the positions of residues N^974^ and F^990^ are indicated. The amphipathic helix is shown in orange with the side chains in ball-and-sticks representation. Hydrophobic residues are clearly located on one side of the helix, while polar and charged residues are located on the opposite side. The Repeat in ToXins (RTX) domain, which adopts a β-roll structure even in the absence of Ca^2+^ ions, is oriented toward the back of the representation.

The structure of pro-HlyA is unknown and established homology modeling tools such as PHYRE2 (Kelley et al., 2015) were unable to model a complete structure of pro-HlyA. HlyA is acylated at two Lys residues (K564 and K690) in the cytosol of *E. coli* prior to secretion. Only its acylated version forms pores in the host membrane. Consequently, the non-acylated, inactive form is called pro-HlyA. Recent developments, resulting in the program AlphaFold (Jumper et al., 2021) allowed the modeling of the entire pro-HlyA monomer. Even in the absence of Ca^2+^ ions, the characteristic feature of RTX proteins, the β-roll of the GG-repeats (Linhartova et al., 2010) was completely modeled (Figure 1A).

**FIGURE 2:**
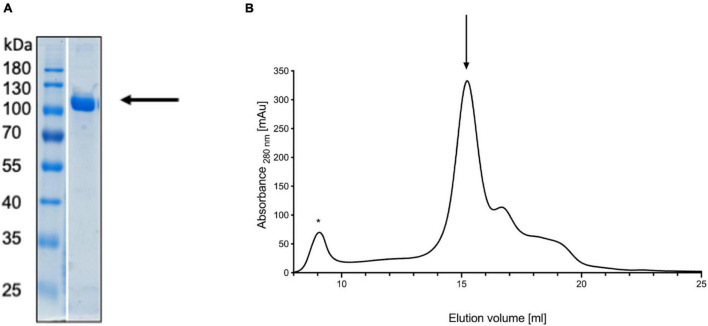
Denaturing gel of refolded pro-HlyA (indicated by an arrow) **(A)** and the corresponding SEC chromatogram **(B)**. The void volume is indicated by an asterisk and the elution peak of pro-HlyA by an arrow. The SDS-PAGE gel was stained with Coomassie Brilliant Blue.

To verify this model experimentally, we turned to small angle X-ray scattering (SAXS). Small angle X-ray scattering allows the study of proteins in solution and offers information about the oligomeric state. Wild type pro-HlyA, i.e., the non-acylated version of the protein, was expressed and purified from inclusion bodies (Figure 2A). As shown in Figure 2B, size exclusion chromatography indicated a broadly eluting sample, which was used for subsequent SAXS experiments.

**FIGURE 3:**
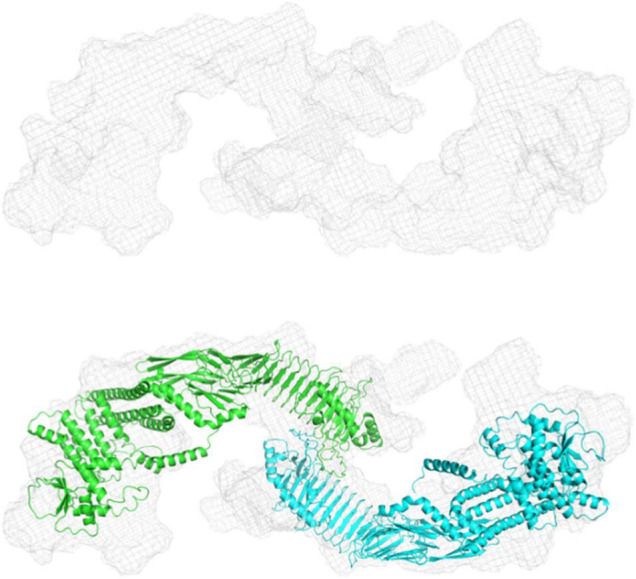
*Ab initio* and rigid body modeling of refolded pro-HlyA. Upper panel: Volumetric envelope of the GASBORMX *ab initio* model. Lower panel: Overlay of the *ab initio* and the dimer model. The single monomers are colored in cyan and green.

We used size exclusion chromatography-coupled SAXS (SEC-SAXS) to separate different higher oligomeric species as well as aggregates from the sample. Analyzing different frames revealed an inhomogeneous distribution within the peak. Frames were subsequently merged using CHROMIX and the corresponding buffer frames were subtracted. The determined molecular weight for pro-HlyA was near to that of the calculated dimer (220.38 kDa), leading to the conclusion that the protein forms a dimer in solution (Table 2). Nevertheless, a monomer/dimer mixture was present in solution and an *ab initio* model for the pro-HlyA dimer (χ^2^:1.19) was calculated using GASBORMX. SASREFMX and the AlphaFold monomer model were used to dock a dimer based on the SAXS data (χ^2^:1.4). With the resulting dimer and the initial monomer, a content of 81.7% dimers and 18.2% monomers in the chosen frames using OLIGOMER was determined. The SAREFMX dimer model was superimposed with the calculated *ab initio* model of GASBORMX and the dimer interface was localized to the C-terminal part of the pro-HlyA protein (Figure 3).

**TABLE 2:** Overall small angle X-ray scattering (SAXS) Data of pro-HlyA.

SAXS Device	BM29, ESRF Grenoble (Pernot et al., 2010, 2013)
**Data collection parameters**	
Detector	PILATUS 1 M
Detector distance (m)	2.869
Beam size	700 μm × 700 μm
Wavelength (nm)	0.099
Sample environment	Quartz capillary,1 mm ø
*s* range (nm^–1^)^‡^	0.025–5.0
Exposure time per frame (s)	2
**Sample**	**pro-HlyA refolded**
Organism	*E. coli* UTI89
UniProt ID and range	P08715
Mode of measurement	Online SEC-SAXS
Temperature (°C)	10
Protein buffer	100 mM HEPES pH 8.0, 250 mM NaCl, 10 mM CaCl_2_
SEC-Column	Superose 6 increase 10/300
Injection volume (μl)	110
Flowrate	0.5 ml/min
Protein concentrations	8.0 mg/ml
**Structural parameters**	
*I(0)* from *P(r)*	97.54
*R*_g_ [real-space from *P(r)*] (nm)	7.04
*I*(0) from Guinier fit	95.91
*s-range* for Guinier fit (nm^–1^)	0.080–0.187
*R*_g_ (from Guinier fit) (nm)	6.65
points from Guinier fit	4–27
*D*_max_ (nm)	25.26
POROD volume estimate (nm^3^)	346.40
**Molecular mass (kDa)**	
From *I(0)*	n.d.
From Qp (Porod, 1951)	242.10
From MoW2 (Fischer et al., 2010)	204.90
From Vc (Rambo and Tainer, 2013)	195.01
Bayesian Inference (Hajizadeh et al., 2018)	208.00
From POROD	173.2–216.5
From sequence	110.19 (monomer) 220.38 (dimer)
**Structure evaluation**	
Gasbor MX fit χ^2^	1.19
Sasref MX fit χ^2^	1.4
Oligomer fit χ^2^ (ratio)	1.32 (81.7% dimer/18.2% monomer)
Ambimeter score	2.525
**Software**	
ATSAS Software Version (Manalastas-Cantos et al., 2021)	3.0.4
Primary data reduction	CHROMIXS (Panjkovich and Svergun, 2017)/PRIMUS (Konarev et al., 2003)
Data processing	GNOM (Svergun, 1992)
*Ab initio* modeling	GASBORMX (Svergun et al., 2001; Petoukhov et al., 2012)
*Rigid body* modeling	SASREFMX (Petoukhov and Svergun, 2005; Petoukhov et al., 2012)
Mixture analysis	OLIGOMER (Konarev et al., 2003)
Superimposing	SUPCOMB (Kozin and Svergun, 2001)
Structure evaluation	AMBIMETER (Petoukhov and Svergun, 2015)
Model visualization	PyMOL (www.pymol.org)

*‡s = 4πsin(θ)/λ, 2θ – scattering angle, λ – X-ray-wavelength, n.d. not determined.*

The nature of the additional densities is highly speculative and might reflect the high flexibility of pro-HlyA. However, the good quality of the main part of the dimer model suggests that the overall structure of pro-HlyA is of high reliability. Most importantly, an amphipathic helix (Figure 1B) covering residues 975–987 within the secretion signal was present in the AlphaFold model.

In the early studies, random and directed mutagenesis methods were applied to the secretion signal of an N-terminally truncated construct of HlyA, called HlyA1 (residue 806–1024; 23 kDa) (Stanley et al., 1991; Kenny et al., 1992; Chervaux and Holland, 1996), which covers three GG-repeats of the RTX domain as well as the secretion signal. These studies revealed that the secretion signal is relatively tolerant toward mutations; however, some mutations had drastic impacts on the amount of secreted HlyA1 and most of them clustered in a proposed amphipathic α-helix predicted between residue L973 and F990 (Koronakis et al., 1989). In light of the AlphaFold model and its fit to the SAXS envelope (Figure 3), we therefore re-investigated the mutational studies of this region (Chervaux and Holland, 1996). It is not expected that pro-HlyA will fold in the cytosol of *E. coli* (Bakkes et al., 2010) as the concentration of free Ca^2+^ is too low [approximately 300 nM (Jones et al., 1999)] to bind to the GG-repeats of the RTX domain and thereby inducing folding. However, secondary structure elements also exist in unfolded proteins as demonstrated by solid state NMR (Curtis-Fisk et al., 2008; Wasmer et al., 2009) making the presence of the amphipathic helix possible in the cytosol of *E. coli*.

**FIGURE 4:**
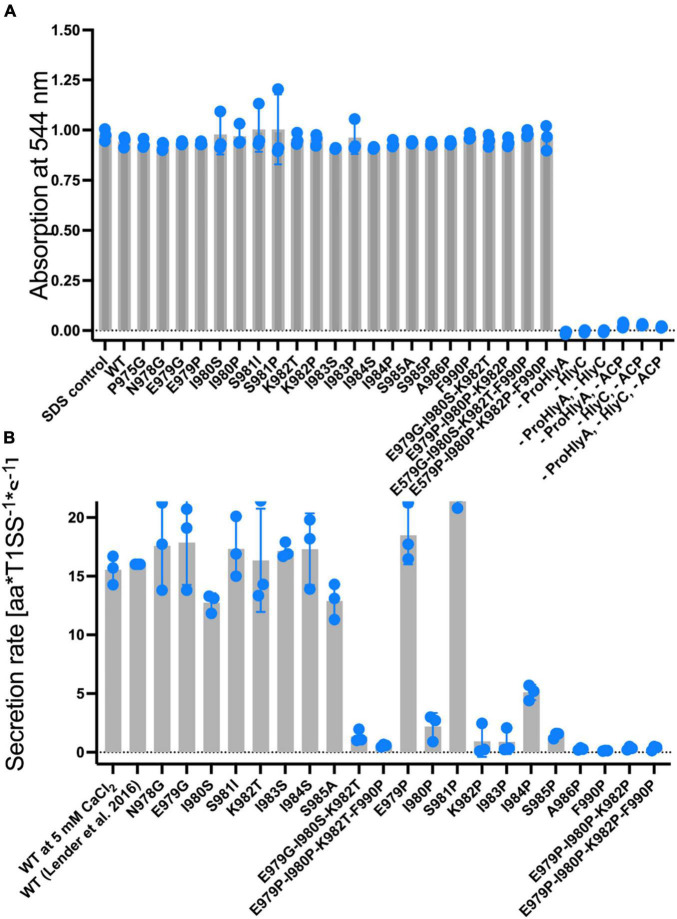
**(A)** Normalized hemolytic activity of wild type HlyA (left bar), single, triple and quadruple mutations within the secretion signal. The lysis of erythrocytes was quantified by measuring the release of hemoglobin by absorption spectroscopy at 544 nm. Control measurements shown to the right of the quadruple mutations lacked HlyA (acylated form), the acylase HlyC, Acyl-carrier protein (ACP), or a combination of these, in the assay. These results demonstrated that lysis was only induced in the presence of acylated HlyA. HlyA is as efficient in hemoglobin release as an SDS incubation (Thomas et al., 2014b) (not shown). Individual assays were performed in at three biological independent experiments and shown as scatter dot plots. **(B)** Summary of the secretion rates of wild type pro-HlyA (left), single, triple and quadruple mutations within the putative secretion signal. The value “WT” was taken from Lenders et al. (2016). Data represent the average of three biologically independent experiments and are shown as scatter dot plots.

We introduced all of these mutations and combinations thereof (Chervaux and Holland, 1996) as well as proline-substitutions into full length HlyA. In a first step, we explored their hemolytic activities (for HlyA) as well as their secretion rates (for non-acylated pro-HlyA). As pro-HlyA requires acylation of two internal lysine residues (K564 and K690) for hemolytic activity (Stanley et al., 1994), wild type and all mutants were activated by an *in vitro* acylation assay according to Thomas et al. (2014b). This allowed us to quantify their activity and, most importantly, normalize it to the amount of HlyA used in the hemoglobin release assay by measuring the absorption spectrum. Here, and in contrast to earlier work (Chervaux and Holland, 1996), normalization to the amount of active HlyA in the assay clearly demonstrated that none of the mutations affected the actual hemolytic activity of HlyA within experimental error (Figure 4A).

The hemolytic activity of all HlyA single point mutants, which were already investigated by Chervaux and Holland (1996), did not affect the hemolytic activity. We also created and included triple mutants since they are part of the predicted amphipathic α-helix to verify whether cumulative effects might be present. As shown in Figure 4A, no change in hemolytic activity was detected for these mutants. Based on the results of the hemolytic assay, we moved one step further and determined the secretion rates of all mutants according to Lenders et al. (2016) (Figure 4B).

In contrast to the hemolytic activity of acylated HlyA, the secretion rates of non-acylated pro-HlyA clearly showed a reduction in the rates for certain mutations. Wild type pro-HlyA was secreted at 14.3 ± 3.1 amino acids*T1SS^–1*^s^–1^, which is in the range of the reported value of 16.0 ± 1.3 amino acids*T1SS^–1*^s^–1^ within experimental error (Lenders et al., 2016). All of the non-proline single point mutations displayed the same secretion rates as the wild type within standard error. The values ranged from 12.8 ± 2.0 amino acids*T1SS^–1*^s^–1^ (I980S) to 20.2 ± 2.1 amino acids amino acids*T1SS^–1*^s^–1^ (E979G). In contrast, all single proline mutations, with the exception of E979P and S981P, displayed a clear reduction in the secretion rates. In the case of E979P and S981P, the rates were slightly higher than the rate of wild type pro-HlyA (19.5 ± 2.3 amino acids*T1SS^–1*^s^–1^) and (19.4 ± 3.4 amino acids*T1SS^–1*^s^–1^, respectively). For the triple (red bars in Figure 4B) and the quadruple (brown bars in Figure 4B) mutants, the secretion rates were close to the background.

**FIGURE 5:**
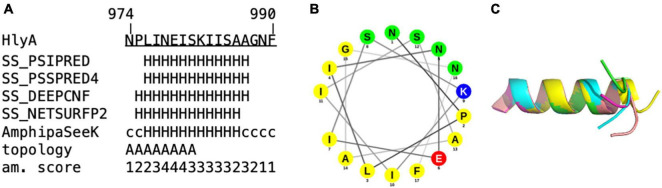
*In silico* analysis of **(A)** amino acids 974 to 990 of the secretion signal of HlyA. H = α-helix, c = coiled coil. Predictions labeled with “SS” were obtained from Quick2D, that utilizes multiple prediction algorithms. Topology = predicted by AmphipaSeeK: “A” indicated those residues that are predicted to be inserted parallel into the membrane. Am. score = amphipathy score predicted by AmphipaSeeK with 1 = lowest amphipathy and 5 = highest amphipathy. **(B)** Helical wheel projection of residue 974–990 of HlyA. Non-polar residues are colored yellow, lysine blue, glutamate red and polar residues green. **(C)** Superimposition of five PEP-FOLD3 models of residue 974–990 of HlyA. The helix is similar in all five models with the C-terminal tail showing variability in orientation.

**FIGURE 6:**
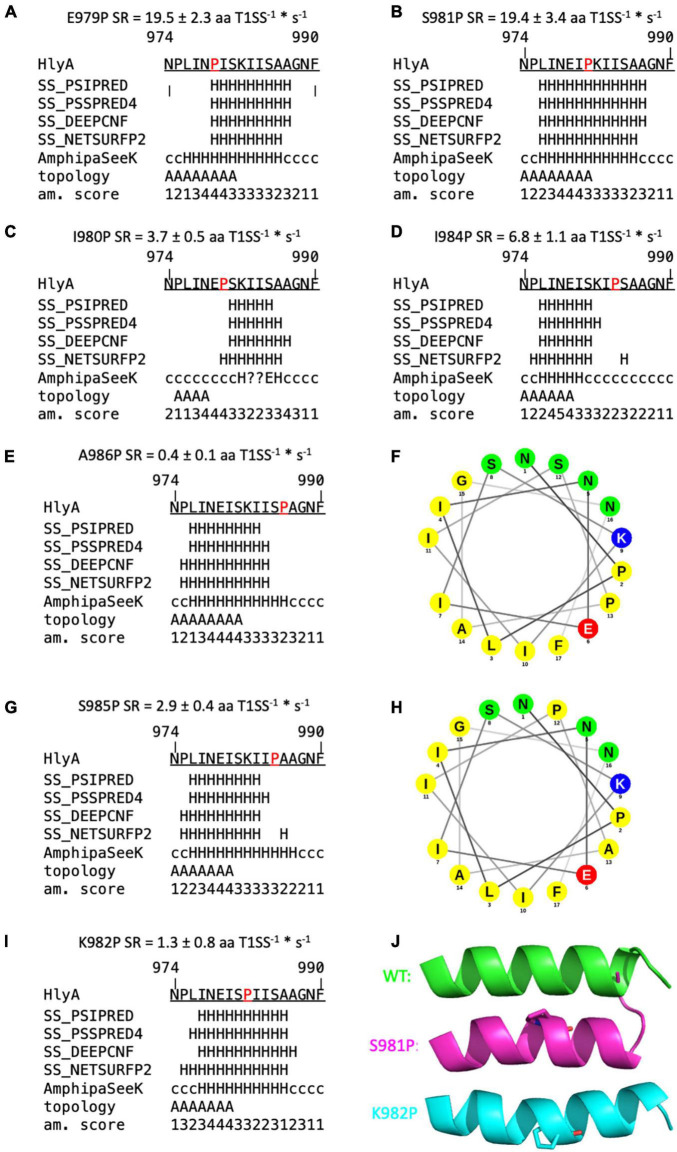
Secondary structure predictions of mutants of the secretion signal of HlyA. Predictions labeled with “SS” are derived from Quick2D. Topology = predicted by AmphipaSeeK, “A” indicated those residues that are predicted to be inserted parallel into the membrane. Am. score = amphipathy score predicted by AmphipaSeeK with 1 = lowest amphipathy and 5 = highest amphipathy. H = α-helix, c = coiled coil, E = β-sheet, ? = no prediction. Mutated residues are marked in red. Secretion rate (SR) is given for each mutant as mean ± SD of three independent measurements. **(A,B)** Single proline mutations with SR similar to wild type pro-HlyA. **(C,D)** Single proline mutations with reduced SR compared to the wild type protein. **(F,H)** Helical wheel projection of A986P **(F)** and S985P **(H)**. Non-polar residues are colored yellow, lysine blue, glutamate red and polar residues green. Proline at position 985 reduces the polarity on the polar site of the amphipathic α-helix compared to wild type HlyA (Figure 1B). **(E,G,I)** Proline substitutions with drastically reduced SR. **(J)** Cartoon representation of PEP-FOLD3 models of wild type pro-HlyA (green), S981P (pink) and K982P (cyan). Mutated proline residues are shown as sticks. All models have an identical orientation for comparison. K982P and S981P bend the helix in opposite directions.

Proline is known as a so-called helix breaker, due to its unique conformation and rigid rotation. Its preferred position in helices is at the N-terminus (Richardson and Richardson, 1988), but helices with proline in or close to the center are still possible (Kim and Kang, 1999). In order to correctly interpret the secretion rates of especially the proline mutants, secondary structure prediction tools (Sapay et al., 2006; Zimmermann et al., 2018) as well as peptide modeling with the tool PEP-FOLD3 (Thevenet et al., 2012) were employed. This was necessary as the algorithm implemented in AlphaFold was not trained on single mutants and is insensitive to single side chain changes (Jumper et al., 2021). Firstly, this analysis revealed that a putative amphipathic α-helix of HlyA is situated between residue P975 and A987 (Figure 5) in strong agreement with the structural model (Figure 1B). Alternatively, the prediction tools placed the α-helix between residues 975 and 987 or 974 and 986. Secondly, the mutants E979P and S981P, which showed secretion rates similar to the wild type within standard deviation (Figure 3), are still able to form an amphipathic α-helix. In contrast, mutants such as I980P and I984P, whose secretion rates were strongly reduced, showed much shorter helices in the predictions (Figure 6).

The secondary structure prediction tools predicted impairments of the amphipathic α-helix for almost all mutants that exhibited a reduced secretion rate. Four mutants were identified whose secretion rate was strongly reduced, but a helix was still predicted: F990P, which is not part of the amphipathic α-helix, K982P, S985P and A986P. However, their secretion rate phenotypes can be rationalized with the help of additional *in silico* tools.

The latter two mutations, S985P and A986P, showed a slightly shortened amphipathic α-helix in the predictions (Figures 6E,G) while A986 marks the end of the amphipathic α-helix in wild type pro-HlyA, followed by another Ala residue and a Gly residue. This region [(S)AAG] is therefore flexible, which is also reflected by the five different models from PEP-FOLD3 for wild type HlyA- amphipathic α-helix, where tails project in different directions (Figure 5C). This flexibility is most likely impaired when a proline residue is introduced at this position, which explains the observed reduced secretion rate. In addition to the reduced flexibility, the polarity of the polar side of the amphipathic α-helix is reduced for S985P, which is illustrated in the helical wheel projection (Figure 6H).

The mutant K982P also results in a change of polarity, correlating with a reduced secretion rate of 1.3 ± 0.8 aa T1SS^–1*^s^–1^ (Figure 6I). However, the mutant K982T, which equally eliminates the positive charge at this position, shows wild type-like secretion (Figure 4B), showing that a positive charge at this position is not essential for efficient secretion. However, a proline at this position introduces a bend to the amphipathic α-helix as seen in the PEP-FOLD3 models (Figure 6J). The proline substitution at n-1 (S981P) also shows a bend of the amphipathic α-helix but no impairment of the secretion rate within experimental error (19.4 ± 3.4 aa T1SS^–1*^s^–1^) (Figure 6J). These two mutants were found to bend the amphipathic α-helix in opposite directions, with S981P resembling the wild type more than K982P (Figure 6I). This is in line with the secretion rates (Figure 4B) and further supports the hypothesis that the precise secondary structure of this motif is essential for secretion.

F990 is not part of the predicted amphipathic α-helix but highly susceptible to mutations and essential for secretion. In previous studies it has been demonstrated that a substitution of this residue to His, Cys, Ala, Ser, Ile, Asn or Pro strongly reduced the secretion of HlyA to <20% compared to wild type (Chervaux and Holland, 1996). The substitution to Tyr was least affected and allowed a secretion of approximately 35% compared to wild type protein (Chervaux and Holland, 1996). Interestingly, CyaA from *B. pertussis* also contains a Tyr residue at this position (Bumba et al., 2016).

Further support of the importance of the amphipathic α-helix comes from calculations of the hydrophobic moment (Table 3). The hydrophobic moments decreases with decreasing secretion rates with the only exception being the I984P mutant. This dependence again highlights that the amphipathic α-helix plays an essential role during secretion.

**TABLE 3:** Hydrophobic moments of the single side mutations of the predicted amphipathic α-helix.

Sequence	Calculated hydrophobic moment	Secretion rate [aa/T1SS*sec]
NPLINPISKIISAAGNF	0.519	19.5
NPLINEISKIISAAGNE	0.494	16
NPLINEIPKIISAAGNE	0.492	19.4
NPLINEISKIIPAAGNE	0.481	2.9
NPLINEISKIPSAAGNE	0.436	6.8
NPLINEPSKIISAAGNE	0.420	3.7
NPLINEISKIISPAGNE	0.41	0.4
NPLINEISPIISAAGNE	0.403	1.3

*Hydrophobic moments were calculation using hmoment (https://www.bioinformatics.nl/cgi-bin/emboss/hmoment) employing standard settings. Mutants are arranged according to decreasing hydrophobic moments.*

**FIGURE 7:**
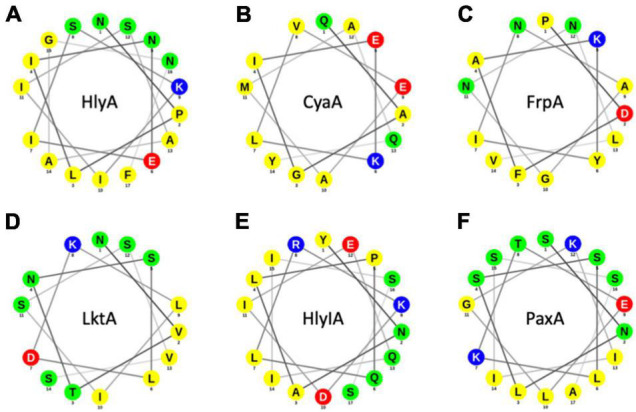
Helical wheel projections of RTX proteins that can be secreted by the HlyBD-TolC apparatus. Helical wheels were drawn with NetWheel. Non-polar residues are shown in yellow, polar residues in green, basic residues in blue and acidic residues in red. Next to HlyA **(A)**, secretion by HlyBD-TolC has been shown for CyaA **(B)** by Masure et al. (1990), for FrpA **(C)** by Thompson and Sparling (1993), for LktA **(D)** by Highlander et al. (1990), for HlyIA **(E)** by Gygi et al. (1990) and for PaxA **(F)** by Kuhnert et al. (2000).

At least five other RTX proteins can be secreted by the HlyBD-TolC system (Figure 7; Gygi et al., 1990; Highlander et al., 1990; Masure et al., 1990; Thompson and Sparling, 1993; Kuhnert et al., 2000). The structure of the C-terminus of one of these has been solved (CyaA), and also shows an amphipathic α-helix followed by an aromatic residue (Bumba et al., 2016). The secondary structures of the last 60 residues of the remaining four RTX proteins have been predicted with secondary structure prediction tools and all four show amphipathic α-helices in the N-terminus of their secretion signal followed by an aromatic residue (Figure 7). Taken together, the secretion rate phenotypes of the HlyA mutants in combination with their *in silico* analysis and the comparisons to heterologous substrates of the HlyBD-TolC system strongly support the presence of an amphipathic α-helix in the secretion signal and emphasize the importance of the correct secondary structure for secretion.

In summary, the data presented here including functional data, structural modeling and *in silico* analysis strongly point toward an essential role of an amphipathic α-helix covering amino acid residues 970-987 in the C-terminal secretion signal of HlyA.

## Discussion

The AlphaFold algorithm (Jumper et al., 2021) correctly modeled the β-roll domain of pro-HlyA (Figure 1A) even in the absence of Ca^2+^ ions. Currently, ligands cannot be included, but the predicted β-roll of pro-HlyA aligns well with the corresponding regions of alkaline protease (Baumann et al., 1993) or block IV/V of the RTX domain of CyaA (Bumba et al., 2016), increasing the confidence in the model. Motivated by this three-dimensional model produced by AlphaFold (Figure 1A) that demonstrated the presence of an amphipathic α-helix in the secretion signal (Figure 1B), we re-examined the presence and role of this amphipathic α-helix for the secretion of pro-HlyA by its cognate T1SS.

Small angle X-ray scattering (SAXS) data of pro-HlyA in solution were used to further improve the quality and accuracy of the model (Figure 3). One has to note that pro-HlyA in solution predominantly forms dimers (Thomas et al., 2014a), a fact that was also confirmed by SEC-SAXS and included in docking of the pro-HlyA model into the SAXS envelope (Figure 3). Without going into the details of the obtained model, the presence of an amphipathic α-helix (Figure 1B) was already proposed by Koronakis et al. (1989) with slight deviations in the exact position and length of the α-helix. Based on this agreement between theory and experiment, we analyzed the precise nature of the amphipathic α-helix signaling and its involvement in the secretion process of HlyA. We also tried to verify this structure by single particle cryo-EM. The C-terminal part, i.e., the RTX domain and the secretion signal, fitted well into the map. However, no density was observed for the N-terminal part indicating a high degree of flexibility and/or denaturation during grid preparation (not shown).

The hemolytic assay of HlyA and the mutants did not reveal any significant differences in activity (Figure 4A) as long as the proteins were used at identical concentrations. This is in contrast to mutations within the last six amino acids of HlyA (Jumpertz et al., 2010). Here, a reduced hemolytic activity was determined, which likely was due to impaired folding of the mutant protein. In the case of mutations within the amphipathic α-helix, folding and the resulting activity is apparently not influenced, pointing toward a role of these residues in an earlier step of the secretion process. Thus, steps taking place on the extracellular side are not impaired and we focused processes at the cytoplasmic side and measured the secretion rates per transporter of each mutant and compared it to the wild type protein (Figure 4B). All of the non-proline, single mutations within the amphipathic α-helix showed no change in the secretion rate per transporter within experimental error. For the single proline mutations, the situation was more complex. Positions E979 and S981 were insensitive to mutations to proline, while positions I980, K982, I983, I984, S985, and A986 were very sensitive and showed drastic reductions of the secretion rate, some had secretion rates close to background values (Figure 4B). This was also true for position F990, which is not part of the amphipathic α-helix, but one of the few amino acids that were determined in mutational studies to be essential for efficient secretion (Chervaux and Holland, 1996; Holland et al., 2016). Since the most efficient secretion can be achieved by a substitution with another aromatic residue, π-π interactions can be assumed, that are disrupted in F990P. Vernon et al. provided an extensive study analyzing π-π interactions in different protein crystal structures (Vernon et al., 2018). Amongst other findings they show that Phe and Tyr have very similar preferences for the nature of their contacts, and that π-π stacking with non-aromatic residues is actually more common than aromatic-aromatic stacking. Furthermore, they identified Arg as the first or second most likely interaction partner for any given aromatic side chain (Vernon et al., 2018). Conserved Arg residues can be found, for example in the cytosolic domain (CD) of HlyD and could present an interaction partner to F990.

In summary, these results supported the notion that secretion rates as read-out for impaired secretion efficiency is a valid approach. The triple and quadruple mutants were also drastically impaired in their secretion rates. Importantly, the secretion rate of the triple mutant E979G/I980S/K982T, containing no proline residues, was also reduced close to background levels. This was in contrast to the single mutations, which displayed secretion rates identical to the wild type protein, suggesting an additive or even cooperative effect of these mutations in HlyA secretion that disrupted the predicted amphipathic α-helix. Since the AlphaFold algorithm was not trained to take single site mutations into account for accurate structure predictions (Jumper et al., 2021), we turned to an *in silico* analysis of the mutants. For wildtype pro-HlyA, a close match between the model and the prediction for the amphipathic α-helix using different programs was obtained (Figure 5). More importantly, however, was the analysis of the mutants. In all cases, in which the secretion was not impaired within experimental error, an amphipathic α-helix was predicted that resembled strongly the wild type. In contrast, in all cases that impaired the secretion rates, the length of the amphipathic α-helix was reduced (Figures 6C,D) or the bending of the amphipathic α-helix was inverted (Figures 6I,J). Thus, the correct length and bending direction are indispensable for efficient secretion of the substrate, which is also supported by the calculation of the hydrophobic moments of the mutants (Table 3).

Moving one step further, we also analyzed further RTX toxins, which have been secreted in the past using the HlyA T1SS (Figure 7). For CyaA, the structure of block IV/V of the RTX domain was determined by X-ray crystallography but the region of the amphipathic α-helix and the flanking aromatic residue (F990 in HlyA) is not resolved (Bumba et al., 2016). Consequently, we performed an *in silico* analysis of those five additional substrates. As shown in Figure 7, all five RTX proteins contained an amphipathic α-helix and a flanking aromatic residue. Obviously, five examples of substrates of sub-family 2 T1SS are not sufficient to make a real significant statement, but these results suggest that the ‘amphipathic α-helix/aromatic residue’ motif might be a general feature of sub-family 2 T1SS and also impose a sort of substrate selectivity. Overall, we propose that the presence and bending of the amphipathic α-helix combined with a C-terminally flanking aromatic residue triggers an early step in substrate secretion. Eventually it even constitutes the initial trigger to assemble the continues channel across the periplasm, through which HlyA is transported in one-step into the extracellular space.

## Data Availability Statement

The raw data supporting the conclusions of this article will be made available by the authors, without undue reservation. SAXS data were uploaded to the Small Angle Scattering Biological Data Bank (SASBDB) (Kikhney et al., 2020), with the accession codes SASDM67.

## Author Contributions

LS and SS conceived and directed this study. SP, KK, and IE conducted the expression and protein purification. SP performed the hemolytic assays. ML determined the secretion rates. JR generated the SAXS model of HlyA. OS and KK conducted the bioinformatic analyses. MM and BL performed the single particle cryo-EM experiments including data evaluation. OS, IE, KK, JR, SS, and LS wrote the manuscript. All authors read and approved the manuscript.

## Conflict of Interest

The authors declare that the research was conducted in the absence of any commercial or financial relationships that could be construed as a potential conflict of interest.

## Publisher’s Note

All claims expressed in this article are solely those of the authors and do not necessarily represent those of their affiliated organizations, or those of the publisher, the editors and the reviewers. Any product that may be evaluated in this article, or claim that may be made by its manufacturer, is not guaranteed or endorsed by the publisher.

## Footnotes

1
https://colab.research.google.com/github/sokrypton/ColabFold/blob/main/AlphaFold2.ipynb

2
www.pymol.org
